# Ten Reasons Why Abdominal Bandages Should Not Be Used after Labor

**Published:** 1893-11

**Authors:** W. B. Conway

**Affiliations:** Athens, Ga.; Late Physician to the Virginia Agricultural and Mechanical College


### TEN REASONS WHY THE ABDOMINAL BANDAGE SHOULD NOT BE USED AFTER LABOR.

BY W. B. CONWAY, M. D. ATHENS, GA.

Late Physician to the Virginia Agricultural and Mechanical College.

In this day of antiseptics in surgical as well as obstetrical practice, no one will deny the importance of guarding every avenue of infection. I do not mean to say that the rules laid down are infallible, and should be carried out in every case. There are exceptions, as in all cases, and the experienced, common sense physician will distinguish at once the pathological conditions requiring the use of the bandage. My experience teaches me that the latter are exceptional cases and are few and far between. The urgent calls for the bandage are made upon the accoucheur, especially by young women in the higher walks of social life, basing their reasons upon the same flimsy pretext that many of them do with regard to the corset. Hence I give it as my unqualified opinion that the abdominal bandage is absolutely useless, if not a source of great danger. The idea of many is, that it restores the uterus and the other abdominal organs to their normal condition, preventing displacements of the uterus, antiversion, antiflexion, subinvolution, etc.

RULES.

1st.—It is unnatural.

2d.—It is liable to become soiled and hence a harbor for microbes.

3rd.—It increases irritation of the tired and overworked abdominal organs.

4th.—It interferes with the necessity of frequent antiseptic ablutions.

5th.—It is difficult to keep in place, unless made to order.

6th.—It binds down the weak uterus and promotes the return of a displacement or a subinvolution.

7th.—It predisposes to puerperal infection, disturbing the peripheral and cerebro-spinal centers.

8th.—It increases rather than diminishes the danger of post-partum hemorrhage.

9th.—It prevents digestion, assimilation, and intestinal peristalsis and tends to bladder trouble.

10th.—It is unsafe to apply it by anyone except the accoucheur or an experienced nurse.

